# Social determinants of health disparities in Staten Island compared with Manhattan, Queens, Brooklyn, and the Bronx: Contribution to COVID‐19 outcomes

**DOI:** 10.1002/iid3.1151

**Published:** 2024-01-19

**Authors:** Masha Kolesnikova, Haram Abdelmajid, Stephan Kohlhoff, Tamar A. Smith‐Norowitz

**Affiliations:** ^1^ Department of Pediatrics, Division of Infectious Diseases State University of New York Downstate Health Sciences University Brooklyn New York USA

**Keywords:** Coronavirus disease‐2019, New York, social determinants of health, Staten Island, hospitalization rates, death rates

## Abstract

**Introduction:**

Social determinants of health (SDH) negatively affected Coronavirus disease‐2019 (COVID‐19) outcomes within the five boroughs of New York City. The goal of this study was to determine whether differences in social demographics within the borough of Staten Island, compared with the other four boroughs, may have contributed to poor COVID‐19 outcomes in Staten Island.

**Methods:**

Data were obtained from public data sources. Social demographics obtained included age, household income, poverty status, and education level. COVID‐19 infection, hospitalization, and death rates reported from Staten Island were compared with rates from Manhattan, Queens, Brooklyn, and the Bronx (February 29, 2020–October 31, 2022). Mean differences in case rates of COVID‐19 were higher in Staten Island compared to all four boroughs.

**Results:**

Mean differences in hospitalization and death rates were higher than Manhattan but similar to the other four boroughs. Within Staten Island, case rates were highest in zip codes 10306 and 10309. Hospitalization and death rates were highest in Staten Island zip code 10304. We found that the zip codes of Staten Island with poorer COVID‐19 outcomes had more individuals with less than a high school degree, lower mean household income, higher proportion of households earning less than $25,000 a year, and a greater proportion of individuals using public transportation.

**Conclusion:**

Differences in COVID‐19 infection, hospitalization, and death rates exist between the five boroughs and between the 12 zip codes within Staten Island. These differences in COVID‐19 outcomes can be attributed to different SDH.

## Graphical abstract

Our report presents the findings of COVID‐19 outcomes between the five boroughs of New York City, finding that Staten Island, a relatively affluent borough, had poor COVID‐19 outcomes. Each zip code of Staten Island was analyzed for COVID‐19 outcomes and social demographics in the form of social determinants of health were evaluated. Zip codes with more individuals with less than a high school degree, lower mean household income, and a higher proportion of households earning less than $25,000 a year had poorer COVID‐19 outcomes. We believe that this report will be of particular interest to your readership, as it provides COVID‐19 outcome analysis at the level of zip codes within Staten Island, suggesting differences in social determinants of health between various zip codes may explain the poor COVID‐19 outcomes of the borough.

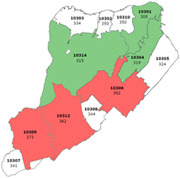

## 1 INTRODUCTION

Coronavirus disease‐2019 (COVID‐19), caused by severe acute respiratory syndrome coronavirus 2 (SARS‐CoV‐2), was first reported in Wuhan, China, in December 2019.^1^ The World Health Organization (WHO) declared a Public Health Emergency of International Concern (PHEIC) on January 30, 2020 and later declared a pandemic (March 11, 2020).^2^ The first case of COVID‐19 in the United States was confirmed in Snohomish County, Washington (January 20, 2020).^3^ The first confirmed case was reported in New York City (NYC) on February 29, 2020.^4^ As numbers of COVID‐19 cases increased, NYC became an epicenter of disease, with cases reported in all five boroughs: the Bronx, Brooklyn, Manhattan, Queens, and Staten Island.^5^

Each borough has a unique set of demographic and socioeconomic characteristics, with variation between different neighborhoods within a borough. Initial studies from 2020 reported COVID‐19 case rates, hospitalizations rates, and death rates varied significantly across the five boroughs.^6^, ^7^ The differences in COVID‐19 reporting within the five boroughs may be attributable to the social determinants of health (SDHs). The SDHs are defined as conditions in the places where people live, learn, work, and play that affect a wide range of health and quality‐of life‐risks and outcomes including but not limited to socioeconomic class and political and cultural factors.^8^

Of notable interest, Staten Island, considered a relatively affluent borough,^9^ was a hotspot for COVID‐19 disease as of April 13, 2020^9^; the highest average case counts were reported in Staten Island (1578–2258/100,000) compared with the other boroughs (Manhattan, Queens, the Bronx and Brooklyn) (553–962/10,000; 1242–1726/100,000; 1462–2042/100,000; 902–1254/100,000, respectively).^10^ Staten Island also was the only borough that reported case rates higher than 1380/100,000 within each zip code, whereas the other boroughs had zip codes with case rates as low as 300/100,000.^10^ Public online data reported SDHs and COVID‐19 outcomes in NYC; however, it remains unclear whether SDHs constitute a causative relationship with COVID‐19 outcomes within the different boroughs. Staten Island ranked higher than the other boroughs regarding different SDH factors (e.g., education level, poverty status, household income).^9^ However, those factors may not have been equally distributed among the different neighborhoods/zip codes within Staten Island.

To address this knowledge gap, we evaluated whether distributions of SDHs—education level, poverty status, household income, mode of transportation—affected numbers of COVID‐19 cases, hospitalization rates, and death rates in individual neighborhoods (e.g., zip codes) of Staten Island compared with the other boroughs (March 2020–October 2022). In addition, we sought to determine whether differences in social disparities exist within one borough, (e.g., Staten Island and the other boroughs). These differences may have played a distinct role for the higher number of case rates reported in Staten Island.

## 2 METHODS

This was a retrospective study. Social and clinical demographic characteristics were obtained from a nationwide cohort design including all individuals residing in Staten Island, Manhattan, Queens, the Bronx, and Brooklyn, using public city‐wide registries.^11^, ^12^ Inclusion criteria included SARS‐CoV‐2 infections, hospitalization rates, and death rates, due to SARS‐CoV‐2, between February 29, 2020 and October 31, 2022.^12^ Borough‐wide social demographics of interest were collected from the 2019 American Community Survey (ACS) profile report^11^ and the United States Postal Service, US Census Bureau and included age, sex, race, household income, poverty status, and education level.^13^ Each neighborhood was defined by a residential area corresponding to a zip code. No individual‐level data were involved in the analysis. The ACS is a source of information about America's changing population; ACS data are collected primarily over the internet through self‐administered questionnaire. The data from this study was obtained from online public databases and was exempt from IRB approval. Hospitalized was defined as taking someone to the hospital and keeping them there for treatment. Fatality was defined as an occurrence of death due to war, accident or disease.

**Figure 1:**
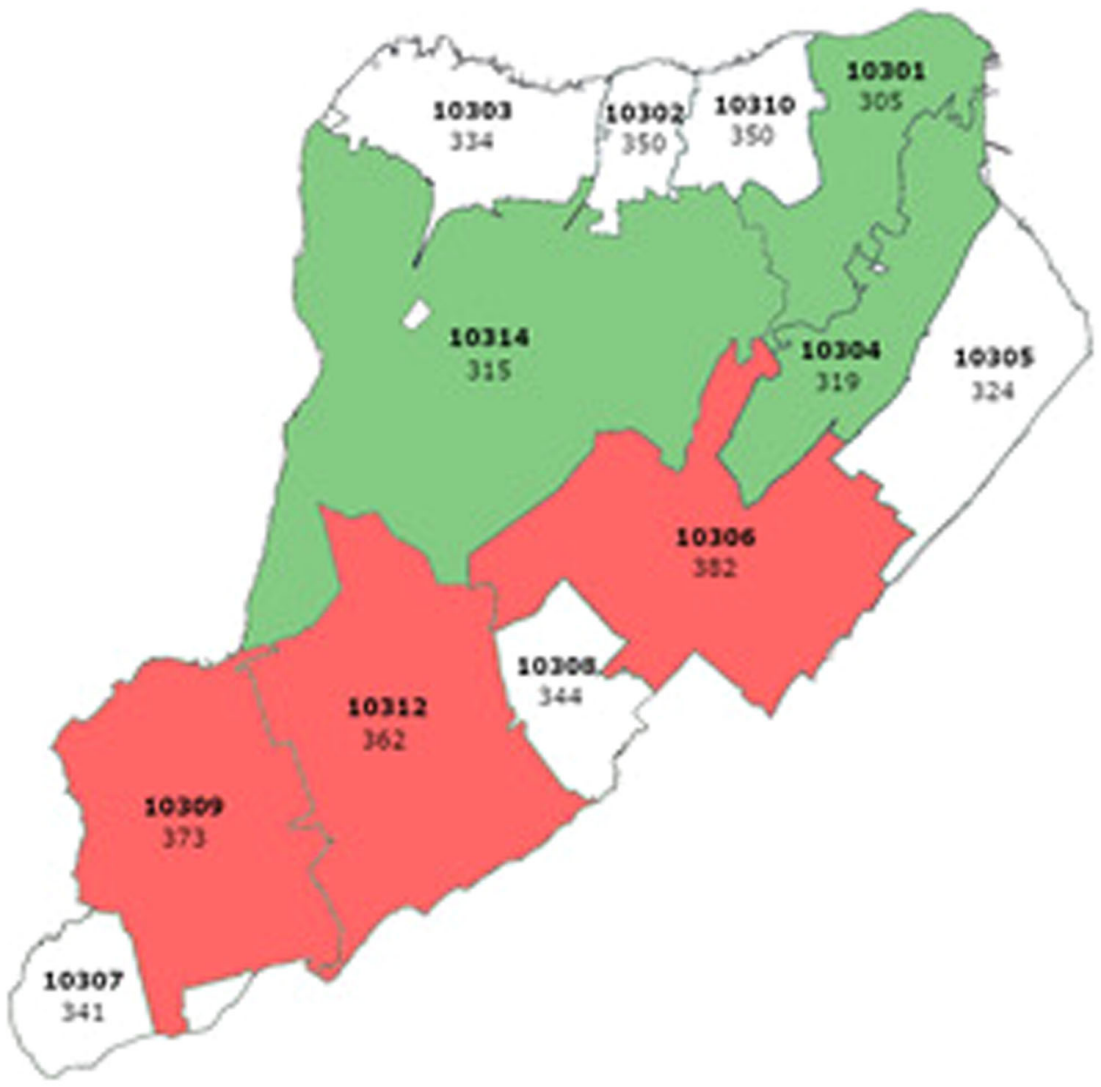
Mean case rates per 100,000 people in the different Staten Island zip codes (2020–2022). The three zip codes with the highest case rates are colored red and the three with the lowest case rates are colored green. Zip codes are written in bold letters. Rates are located below the zip code. *Source*: New York State Department of Health, accessed December 22, 2022.^14^

**Figure 2:**
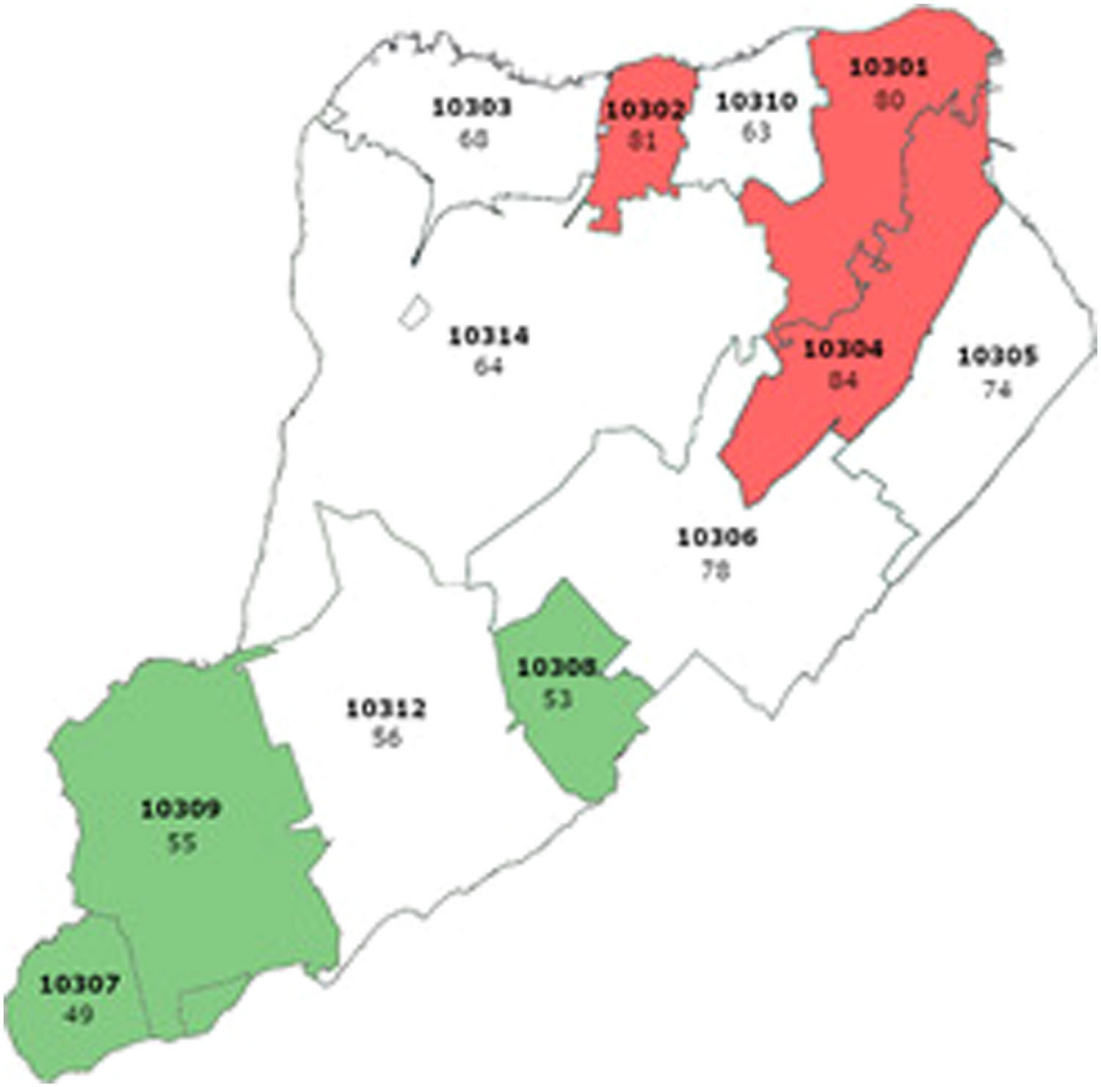
Mean hospitalization rates per 100,000 people in the different Staten Island zip codes (2020–2022). The three zip codes with the highest hospitalization rates are colored red and the three with the lowest hospitalization rates are colored green. Zip codes are written in bold letters. Rates are located below the zip code. *Source*: New York State Department of Health, accessed December 22, 2022.^14^

**Figure 3:**
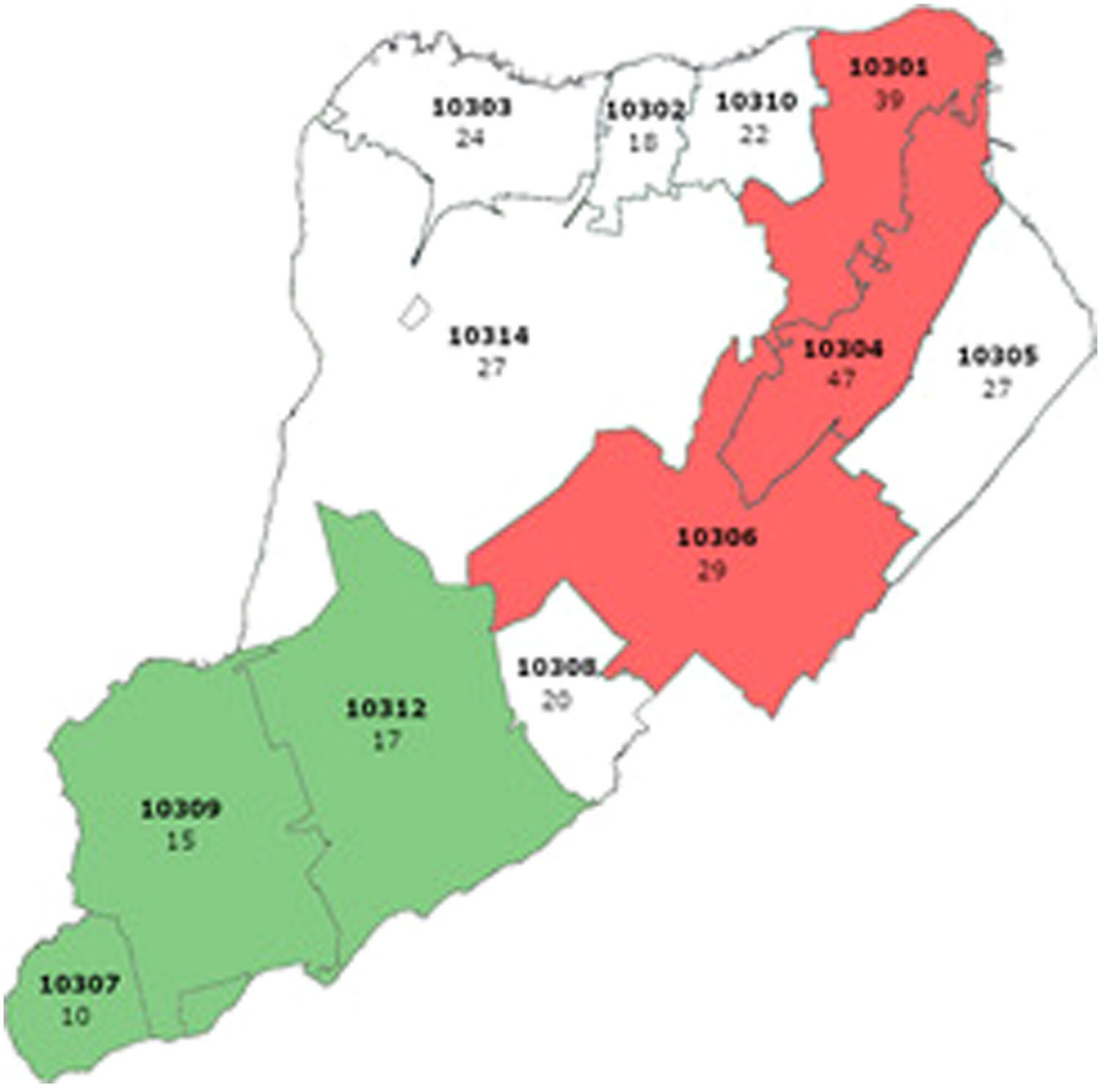
Mean death rates per 100,000 people in the different Staten Island zip codes (2020–2022). The three zip codes with the highest death rates are colored red and the three with the lowest death rates are colored green. Zip codes are written in bold letters. Rates are located below the zip code. *Source*: New York State Department of Health, accessed December 22, 2022.^14^

All statistical tests were calculated using SAS v9.4.3 (SAS Institute). A *p*‐value of <.05 was considered statistically significant. One sample Student's *t*‐test was used to calculate means of case, hospitalization and fatality rates in different boroughs as well as in the different zip codes of Staten Island. Wilcoxon tests were used to calculate mean differences in case, hospitalization, and death rates between Staten Island and other boroughs and between different zip codes because the data distribution was skewed. Data presented in the maps (Figures 1, 2, 3)^14^ were plotted using Stata software (StataCorp).

## 3 RESULTS

1. **Demographics**. Social demographics of each borough and individual Staten Island zip codes are shown in Table 1 (ACS 2019)^11^ and Table 2 (United States Postal Service, US Census Bureau), respectively.^13^ Demographics include income levels, education rates, age, and gender.
2. **Case rates**. The mean number of case rates per 100,000 (2020–2022) for each borough are listed in Table 3. Case rates reported are for Staten Island, the Bronx, Brooklyn, Queens, Manhattan, and city‐wide (340 ± 541, 261 ± 529, 262 ± 449, 271 ± 486, 256 ± 428, 268 ± 470, respectively) (Student's *t*‐tests).
  The mean differences in case rates of COVID‐19 were statistically higher in Staten Island compared with the Bronx (79 ± 78, *p* < .001), Brooklyn (78 ± 119, *p* < .001), Queens (69 ± 78, *p* < .001), Manhattan (84 ± 195, *p* < .001), and city wide (72 ± 97, *p* < .001)(Wilcoxon test) (Table 4).
3. **Hospitalization rates**. The mean number of hospitalization rates per 100,000 (2020–2022) are listed in Table 3. Hospitalization rates reported are for Staten Island, the Bronx, Brooklyn, Queens, Manhattan, and city‐wide (60 ± 65, 84 ± 104, 66 ± 72, 69 ± 87, 50 ± 58, 67 ± 78, respectively) (Student's *t*‐tests).
  The mean differences in hospitalization rates were higher in Staten Island compared with Manhattan (19 ± 20, *p* < .001), but were similar to the Bronx (−15 ± 47, *p* = .52), Brooklyn (4 ± 21, *p* = .09), Queens (0.4 ± 35, *p* = .02), and city‐wide (2 ± 24, *p* = .6) (Wilcoxon test) (Table 4).
4. **Death rates**. The mean number of death rates per 100,000 (2020–2022) are listed in Table 3. Death rates reported are for Staten Island, the Bronx, Brooklyn, Queens, Manhattan, and city‐wide (18 ± 28, 19 ± 43, 17 ± 37, 18 ± 41, 12 ± 24, 17 ± 36, respectively) (Student's *t*‐tests).
  The mean differences in death rates were higher in Staten Island compared with Manhattan (6 ± 6, *p* < .001), but were similar to the Bronx (−2 ± 18, *p* = .19), Brooklyn (−0.09 ± 11, *p* = .13), Queens (−0.96 ± 15, *p* = .09), and city wide (0.6 ± 10, *p* = .02) (Wilcoxon test) (Table 4).
5. **Infection rates, hospitalization rates, and death rates within Staten Island stratified according to zip codes**. Infection rates, hospitalization rates, and death rates per 100,000 within Staten Island were stratified according to 12 zip codes—10301 (305 ± 512, 80 ± 93, 40 ± 64, respectively), 10302 (350 ± 652, 81 ± 81, 18 ± 35, respectively), 10303 (335 ± 628, 68 ± 74, 23 ± 36, respectively), 10304 (320 ± 539, 85 ± 87, 48 ± 77, respectively), 10305 (325 ± 513, 75 ± 80, 28 ± 36, respectively), 10306 (383 ± 573, 79 ± 68, 30 ± 32, respectively), 10307 (341 ± 499, 50 ± 48, 11 ± 25, respectively), 10308 (345 ± 511, 54 ± 49, 21 ± 22, respectively), 10309 (373 ± 550, 58 ± 52, 16 ± 20, respectively), 10310 (351 ± 618, 64 ± 58, 23 ± 40, respectively), 10312 (362 ± 554, 56 ± 50, 17 ± 17, respectively), 10314 (316 ± 499, 65 ± 61, 27 ± 34, respectively) (Student's *t*‐tests) (Table 5).
  The most cases reported were in zip codes 10306 and 10309 (383 ± 573, 373 ± 550, respectively), in the New Dorp (10306) and Prince's Bay (10309) neighborhoods (Table 5).^14^ These two zip codes are located in the central and southern sections of Staten Island, respectively (Figure 1). Mean differences in case rates were compared between zip code 10306 and the other zip codes in Staten Island and were found to be higher (*p* < .05), except in zip code 10309 (*p* = .21) (Wilcoxon test) (Table 6). Mean differences in case rates were compared between zip code 10309 and the other zip codes in Staten Island and were found to be higher (*p* < .05), except in zip code 10306 (*p* = .21) and in zip code 10312 (*p* = .21)(Wilcoxon test) (Table S1). Zip code 10312 is located in the southern section of Staten Island, between zip codes 10306 and 10309 (Figure 1).
  The most hospitalizations reported were in zip code 10304 (85 ± 87), in the Stapleton neighborhood (Table 5).^14^ This zip code is located in the north‐eastern section of Staten Island (Figure 1). Mean differences in hospitalization rates were compared between zip code 10304 and the other zip codes in Staten Island and were found to be higher (*p* < .05) except in zip codes 10301 (5 ± 28, *p* = .14), 10302 (3 ± 24, *p* = .46), and 10306 (6 ± 37, *p* = .47) (Wilcoxon test) (Table S2). Zip codes 10301 and 10302 are located in the northern section of Staten Island while 10306 is located in the central section of Staten Island (Figure 2).
  The most deaths reported were in zip code 10304 (48 ± 77), in the Stapleton neighborhood (Table 5). Mean differences in death rates were compared between zip code 10304 and the other zip codes in Staten Island and were found to be higher (*p* < .05) except in zip codes 10301 (14 ± 22, *p* = 0.084), 10303 (41 ± 68, *p* = .06), 10306 (19 ± 56, *p* = .24), 10307 (52 ± 121, *P* = 1), and 10310 (63 + 89, *p* = .25) (Wilcoxon test) (Table S3). Zip codes 10303 and 10310 are located in the northern section of Staten Island while zip codes 10306 and 10307 are located in the central and southern sections of Staten Island, respectively (Figure 3).
6. **Social demographics within Staten Island stratified according to zip codes**. Social demographics, including age, race, sex, education level, employment status, mean household income, and mode of transportation, per 100,000 within Staten Island were stratified according to 12 zip codes—10301, 10302, 10303, 10304, 10305, 10306, 10307, 10308, 10309, 10310, 10312, and 10314 (Table S4).

**Table 1:** Social demographics/social determinants of health of the five New York City boroughs as well as city‐wide demographics.

	City wide	Manhattan	Bronx	Brooklyn	Queens	Staten Island
Age, years (%)						
Less than 19	23	16	27	25	22	24
20–44	38	43	36	38	35	32
45–65	24	23	24	23	27	27
65 and up	15	17	13	14	16	17
Sex (%)						
Male	48	47	47	47	49	49
Female	52	53	53	53	51	51
Race (%)						
White, non‐Hispanic	34	51	16	38	29	62
Black, non‐Hispanic	20	14	26	27	15	9
Hispanic or Latino	22	22	35	15	21	15
Asian and other	24	13	24	19	34	13
Education (%)						
Less than high school	12	8	21	12	13	8
High school graduate	60	54	62	60	62	65
Bachelor's degree or higher	28	38	17	28	25	27
Employment status (%)						
Employed civilians	61	65	54	61	61	59
Unemployed civilians	3	3	5	3	3	2
Not in labor force	36	32	41	36	36	38
Insurance status, population < 65 (%)						
With insurance	92	95	92	93	90	95
Without insurance	8	5	8	7	10	5
Poverty status (*n*)						
Persons below poverty	16,035	14,039	26,368	17,662	10,821	8271
Mean household income ($)	110,101	170,453	59,664	99,472	96,631	113,245
Household income (%)						
Less than $25,000	22	20	35	22	16	13
$25,000–$74,999	31	22	38	32	35	31
$75,000–$149,999	26	24	20	26	31	31

*Note*: Data collected from the 2019 American Community Survey.^11^

**Table 2:** Social demographics/social determinants of health of the 12 Staten Island zip codes.

	10301	10302	10303	10304	10305	10306	10307	10308	10309	10310	10312	10314
Age (%)												
Less than 19	26	33	33	29	24	24	23	26	28	31	25	24
20–44	36	39	37	34	35	31	37	34	37	34	32	32
45–65	26	19	23	27	28	29	30	24	24	26	30	29
65 and greater	12	10	7	9	13	16	10	16	11	10	13	15
Race (%)												
White, non‐Hispanic	57	52	35	49	78	90	95	95	92	56	92	78
Black, non‐Hispanic	26	22	39	30	4	2	1	1	2	24	1	4
Asian	7	4	8	10	11	5	3	3	4	6	5	14
Other	11	22	17	11	6	4	2	2	2	13	2	4
Sex (%)												
Male	49	50	50	48	49	48	8	49	50	54	49	48
Female	51	50	50	52	51	52	92	51	50	46	51	52
Education status (%)												
Less than high school	12	16	14	15	11	7	6	7	7	9	7	8
High school graduate	63	67	68	63	65	70	69	71	70	69	69	69
Bachelor's degree or higher	25	17	18	22	24	23	24	22	24	22	24	23
Employment status (%)												
Employed	59	58	58	59	57	58	64	63	62	60	62	60
Unemployed	41	42	42	41	43	42	36	37	38	40	38	40
Mean household income ($)	75,825	54,764	48,640	73,771	65,602	76,625	102,903	83,296	93,266	67,168	87,692	76,432
Household income (%)												
Less than $25,000	25	28	28	24	20	17	14	11	11	25	13	15
$25,000–$59,999	27	26	27	30	24	21	19	20	22	24	21	24
$60,000–$149,999	37	35	37	34	43	43	44	46	44	36	46	44
Greater than $150,000	11	11	7	12	13	19	23	23	23	15	20	17
Means of transportation to work (%)												
Self‐transport (car, motorcycle, walk, etc.)	55	64	62	55	62	69	81	70	74	65	76	71
Public transport/taxicab	42	34	36	42	36	29	17	29	24	33	22	26

*Note*: Data collected from the United States Postal Service, US Census Bureau.^13^

**Table 3:** Case rates, hospitalization rates, and death rates due to COVID‐19 in the five boroughs of New York City and city wide.

Borough	Mean case rate (SD)	Mean hospitalization rate (SD)	Mean death rate (SD)
City wide	267.6 (469.8)	66.9 (77.7)	16.6 (35.8)
Bronx	261 (529)	84 (103.9)	18.7 (43)
Brooklyn	261.9 (449)	65.7 (72)	17 (36.7)
Manhattan	256 (427.8)	50 (57.6)	11.6 (23.7)
Queens	271 (485.7)	68.9 (87)	18 (40.7)
Staten Island	340 (540.7)	60 (65)	17.7 (27.9)

*Note*: Data represented as mean + standard deviation (SD)/10,000 people (2020–2022) (Student's *t*‐tests).

**Table 4:** Case rates, hospitalization rates, and death rates due to COVID‐19 in Staten Island compared with the other four boroughs of New York City and city wide.

Comparison borough	Mean diff case rate (SD)	*p*‐Value	Mean diff hospitalization rate (SD)	*p*‐Value	Mean diff death rate (SD)	*p*‐Value
City wide	72 (97)	<.0001	2 (24)	.0569	0.6 (10)	.0184
Bronx	79 (77.5)	<.0001	−14.8 (46.6)	.5191	−1.5 (18)	.1883
Brooklyn	78 (119)	<.0001	3.7 (20.7)	.0899	−0.09 (11)	.1294
Manhattan	84 (195)	<.0001	19 (19.8)	<.0001	5.7 (6)	<.0001
Queens	68.8 (78)	<.0001	0.4 (35)	.0229	− 0.96 (15)	.0919

*Note*: Data represented as mean differences + standard deviation (SD). A *p*‐value of <.05 was considered statistically significant (Wilcoxon test).

**Table 5:** Case rates, hospitalization rates, and death rates according to different Staten Island zip codes.

Zip code	Mean case rate (SD)	Mean hospitalization rate (SD)	Mean death rate (SD)
10301	305 (512)	80 (93)	39.5 (63.8)
10302	350 (652)	81.6 (81)	18 (35)
10303	334.8 (628)	68 (74)	23 (35.5)
10304	319.6 (538.6)	84.9 (87)	47.7 (76.5)
10305	324.5 (513)	74.8 (80)	27.8 (35.5)
10306	382.5 (573)	78.8 (68.2)	29.5 (32)
10307	341 (499)	49.6 (48)	10.7 (24.5)
10308	344.8 (511)	53.8 (48.9)	20.6 (22)
10309	373 (550)	58.0 (51.6)	15.5 (20)
10310	350.6 (618)	63.7 (58)	22.9 (40)
10312	362 (554)	56 (50)	17 (17)
10314	315.7 (499)	64.7 (60.7)	27 (34)

*Note*: Data represented as mean + standard deviation (SD)/100,000 people (2020–2022) (Student's *t*‐tests).

**Table 6:** Comparison of case rates between zip code 10306 and other zip codes in Staten Island.

Zip code compared to	Mean difference (SD)	*p*‐Value
10301	77.6 (96.5)	<.0001
10302	32 (126.5)	<.0001
10303	47.7 (112)	<.0001
10304	62.9 (83)	<.0001
10305	58 (85.9)	<.0001
10307	41 (137)	<.0006
10308	37.7 (91)	<.0001
10309	9.5 (115)	.2059
10310	32 (102)	<.0001
10312	20 (89)	<.0015
10314	66.8 (89)	<.0001

*Note*: Data represented as mean differences + standard deviation (SD)/100,000 people (2020–2022). A *p*‐value of <.05 was considered statistically significant (Wilcoxon test).

The three zip codes with the highest case rates (10306, 10309, and 10312) were located in the southern area of Staten Island (Figure 1). The three zip codes with the lowest case rates (10301, 10304, and 10314) were located in the central area of Staten Island (Figure 1).

The three zip codes with the highest hospitalization rates (10301, 10302, and 10304) were located in the northern area of Staten Island (Figure 2). The three zip codes with the lowest hospitalization rates (10307, 10308, and 10309) were located in the southern area of Staten Island (Figure 2). Trends showed that these three zip codes were those with the highest proportion of households with an annual income greater than $150,000 (23,281 per 100,000; 22,620 per 100,000; 22,992 per 100,000; respectively) and the highest proportion of White, Non‐Hispanic inhabitants (93,431 per 100,000; 93,592 per 100,000; 91,058 per 100,000; respectively) (Table S4).

The three zip codes with the highest death rates (10301, 10304, and 10306) were located in the northern and central area of Staten Island (Figure 3). The three zip codes with the lowest death rates (10301, 10304, and 10306) were located in the southern area of Staten Island (Figure 3). Trends showed that these three zip codes were those with the highest mean household incomes ($102,903; $93,266; and $87,692; respectively), the lowest number of individuals with education levels less than high school (5576 per 100,000; 6000 per 100,000; 6124 per 100,000; respectively) (Table S4).

## 4 DISCUSSION

This analysis found that case rates of COVID‐19 were higher in Staten Island compared with the other four boroughs. However, hospitalization rates and death rates in Staten Island were not higher compared with Brooklyn, the Bronx, and Queens. Even though case rates were higher in Staten Island, clinical outcomes were not as high as expected. This could be attributed to more favorable social determinants of health within the population of Staten Island. Within specific zip codes (10307, 10308, 10309, and 10312) of Staten Island, hospitalization and death rates were lower depending on presence of protective SDH (lower proportion of individuals with less than a high school degree, higher mean household income, higher proportion of households with an annual income greater than $150,000).

The distribution of COVID‐19 outcomes, including number of cases, deaths, hospitalizations, tests, and vaccinations across the five boroughs can provide insight into the factors influencing COVID‐19 statistics in different neighborhoods. Prior studies reported variations in COVID‐19 statistics between the boroughs attributable to the SDHs, suggesting communities with negative SDHs were disproportionally affected by COVID‐19.^6^, ^9^, ^10^, ^15^, ^16^ Moten et al. compared the Social Vulnerability Index (SVI), a metric created by the CDC tracking 15 social factors observed to affect health,^17^ of each NYC county—Kings county, Queens county, New York county, Bronx county, and Richmond county—in regard to COVID‐19 cases, deaths, and hospitalizations in March 2020 and May 2020.^9^ Based on the reported SVI, counties were designated low, moderate, or high vulnerability (LV, MV, and HV) with results showing that the average numbers of daily COVID‐19 cases and deaths in the MV and HV counties were greater than those of the LV counties.^9^ Additionally, Wadhera et al. compared the rates of hospitalizations and deaths within each borough up to April 25, 2020 to demographic characteristics such as race, poverty status, and education level.^6^ The findings showed that the most affluent borough, Manhattan, experienced the lowest number of COVID‐19‐related hospitalizations and deaths, whereas the Bronx, which has the highest proportion of racial minorities, the most individuals living in poverty, and the lowest education level, had higher rates of COVID‐19 hospitalizations and deaths.^6^ These findings were further supported by Baidal et al. who similarly found, through May 14, 2020, that neighborhoods with more non‐White individuals, Hispanic individuals, individuals in poverty, or housing crowding had higher proportions of COVID‐19 cases, hospitalizations, and deaths.^16^ De Jesus et al. demonstrated that the SDHs can predict COVID‐19 case and death rates within the five boroughs through July 2021.^15^ Predictors for COVID‐19 case and death rates include proportion of residents over the age of 65, proportion of White residents, and proportion of residents without health insurance.^15^

Of particular interest, Staten Island, considered a relatively affluent neighborhood with an SVI index of LV,^9^ has been shown to be a hotspot of disease.^10^ Maroko et al. evaluated rates of COVID‐19 case rates among the neighborhoods of NYC in comparison to poverty status, race, education level, insurance status, and median household income, finding that Staten Island, despite having a relatively higher proportion of white residents, a relatively higher median household income, and relatively lower unemployment rate, had case rates similar to neighborhoods of the Bronx and Queens with a higher proportion of negative SDHs.^10^ While De Jesus et al. found a number of SDHs predictive of COVID‐19 cases and hospitalizations within Brooklyn, Queens, Manhattan, and the Bronx, no such explanatory variable was identified within Staten Island among 17 SDHs.^15^ Instead, the authors proposed that other factors unique to Staten Island predicted rates of COVID‐19 cases and hospitalizations.^15^

This study aimed to address this knowledge gap, suggesting that it is factors within individual neighborhoods/zip codes, not Staten Island as a whole, that contribute to the high case rates. First, we evaluated the COVID‐19 case rates of each borough individually to determine if Staten Island continued to be a hotspot of disease through the start of the pandemic to October 31, 2022, as previously published research evaluated statistics confined to 2020.^9^, ^10^, ^15^ We found that the mean difference in case rates was statistically higher in Staten Island as compared to the other boroughs. Similar analysis of hospitalization and death rates demonstrated that Staten Island had a statistically higher mean difference in hospitalization and death rates than Manhattan but similar numbers to the Bronx, Brooklyn, and Queens. We propose that the high case rate can be attributed to higher testing rates and testing capabilities provided by a borough with a LV index. Similarly, we propose that the hospitalization rates and death rates were similar to those rates in Manhattan as both are categorized as LV whereas the Bronx, Brooklyn, and Queens are HV boroughs with less access to healthcare resources.

Analysis of different zip codes within Staten Island found that zip codes 10306 (New Dorp) and 10309 (Prince's Bay) had the highest case rates of COVID‐19 whereas zip code 10304 (Stapleton) had the highest hospitalization rates and death rates.^14^ Protective factors decreasing hospitalization rates include a higher proportion of households with an annual income greater than $150,000 and a higher proportion of White, Non‐Hispanic inhabitants. Protective factors decreasing death rates include a higher mean household income and a lower proportion of individuals with education levels less than high school. Geographically, the southern area of Staten Island had better COVID‐19 outcomes, suggesting that people living in those zip codes may be realtively more affluent.

This study has limitations including differences in access to COVID‐19 testing, potentially unreported cases, hospitalizations, and deaths due to SARS‐CoV‐2 and differences in demographics between data collection in 2019 and COVID‐19 measurements 2020–2022. Also, it should be noted that during different time periods, alpha, delta, and/or omicron became the most dominant SARS‐CoV‐2 variant. Other concerns may include circulation of other strains and possible coinfection with other viruses. In addition, there is no individual level data available for us to include in this study. The study is mainly descriptive and may contain potential bias.

The data presented in this study contribute to our understanding of possible disparities during the COVID‐19 pandemic by providing insights into the relationship between distributions of SDH and COVID‐19 outcomes in Staten Island compared with the other boroughs. While Staten Island is one of the more affluent boroughs with more favorable SDH, we identified areas of interest for better targeting COVID‐19 poor outcomes and underlying risk factors. Different zip codes within Staten Island demonstrated higher case rates, most probably due to the different variations of SDH ^10^, ^15^, ^16^ or exposure to disinformation about the disease in Staten Island.^18^ Higher ethnic variability, access to quality medical care, life style, and comorbidities are possible factors in variability in COVID‐19 outcomes within each borough.^19^ Future planning for similar pandemics should prepare for interventions targeting the most vulnerable populations and neighborhoods within each borough.

## AUTHOR CONTRIBUTIONS

*Designing research studies*: Tamar Smith‐Norowitz and Stephan Kohlhoff. *Acquiring data*: Masha Kolesnikova. *Analyzing data*: Haram Abdelmajid. *Writing—original draft preparation*: Masha Kolesnikova. *Writing—review and editing*: Masha Kolesnikova, Tamar Smith‐Norowitz, and Stephan Kohlhoff.

## CONFLICT OF INTEREST STATEMENT

The authors declare no conflict of interest.

## Supporting information

Supplementary information.

Supplementary information.

Supplementary information.

Supplementary information.

## Data Availability

Data are available from the authors upon request.
