# Supplementary material for: Social determinants of health disparities in Staten Island compared with Manhattan, Queens, Brooklyn, and the Bronx: Contribution to COVID‐19 outcomes
Source: Immun Inflamm Dis. 2024 Jan 19;12(1):e1151. doi: 10.1002/iid3.1151 (PMC10797650; doi:10.1002/iid3.1151)
Supplement: Supplementary file 1 — Supplementary information. [file IID3-12-e1151-s001.docx]

**Supplemental Table 1. Comparison of case rates between zip code 10309 and other zip codes in Staten Island.**

| Zip code compared to | Mean difference (SD) | *P-value* |
| --- | --- | --- |
| 10301 | 68 (116) | < 0.0001 |
| 10302 | 22.6 (179) | <0.0001 |
| 10303 | 38 (164) | <0.0001 |
| 10304 | 53 (98.7) | <0.0001 |
| 10305 | 48.5 (138.5) | <0.0001 |
| 10306 | -9.5 (115) | 0.2059 |
| 10307 | 31.8 (79) | <0.0001 |
| 10308 | 28 (92) | <0.006 |
| 10310 | 22 (131) | <0.0001 |
| 10312 | 10.7 (63) | 0.2 |
| 10314 | 57 (110) | <0.0001 |

Data represented as mean differences + standard deviation (SD)/per 100,000 people (2020-2022). A *P value* of < 0.05 was considered statistically significant (Wilcoxon test).
