# Supplementary material for: Social determinants of health disparities in Staten Island compared with Manhattan, Queens, Brooklyn, and the Bronx: Contribution to COVID‐19 outcomes
Source: Immun Inflamm Dis. 2024 Jan 19;12(1):e1151. doi: 10.1002/iid3.1151 (PMC10797650; doi:10.1002/iid3.1151)
Supplement: Supplementary file 2 — Supplementary information. [file IID3-12-e1151-s003.docx]

**Supplemental Table 2. Comparison of hospitalization rates between zip code 10304 and other zip codes in Staten Island.**

| Zip code compared to | Mean difference (SD) | *P-value* |
| --- | --- | --- |
| 10301 | 4.7 (28) | 0.1384 |
| 10302 | 3 (23.7) | 0.456 |
| 10303 | 16.7 (25) | 0.0006 |
| 10305 | 10 (27) | 0.0179 |
| 10306 | 6 (37) | 0.4746 |
| 10307 | 35 (56) | <0.0001 |
| 10308 | 31 (55) | <0.0001 |
| 10309 | 29 (48) | <0.0001 |
| 10310 | 21 (35) | 0.0018 |
| 10312 | 28.9 (52) | <0.0001 |
| 10314 | 20 (34) | 0.0161 |

Data represented as mean differences + standard deviation (SD)/100,000 people. A *P value* of < 0.05 was considered statistically significant (Wilcoxon test).
