# Supplementary material for: Social determinants of health disparities in Staten Island compared with Manhattan, Queens, Brooklyn, and the Bronx: Contribution to COVID‐19 outcomes
Source: Immun Inflamm Dis. 2024 Jan 19;12(1):e1151. doi: 10.1002/iid3.1151 (PMC10797650; doi:10.1002/iid3.1151)
Supplement: Supplementary file 3 — Supplementary information. [file IID3-12-e1151-s002.docx]

**Supplemental Table 3. Comparison of death rates between zip code 10304 and other zip codes in Staten Island.**

| Zip code compared to | Mean difference (SD) | *P-value* |
| --- | --- | --- |
| 10301 | 14 (21.6) | 0.0840 |
| 10302 | 27 (45) | 0.0078 |
| 10303 | 41 (67.7) | 0.0625 |
| 10305 | 27.6 (51) | 0.0273 |
| 10306 | 18.7 (55.6) | 0.2402 |
| 10307 | 54 (121) | 1 |
| 10308 | 33 (74.6) | 0.0391 |
| 10309 | 41 (71) | 0.0117 |
| 10310 | 63 (89) | 0.2500 |
| 10312 | 32.6 (64) | 0.0171 |
| 10314 | 20.8 (42.5) | 0.0029 |

Data represented as mean differences + standard deviation (SD)/ 100,000 people. A *P value* of < 0.05 was considered statistically significant (Wilcoxon test).
