# Supplementary material for: Social determinants of health disparities in Staten Island compared with Manhattan, Queens, Brooklyn, and the Bronx: Contribution to COVID‐19 outcomes
Source: Immun Inflamm Dis. 2024 Jan 19;12(1):e1151. doi: 10.1002/iid3.1151 (PMC10797650; doi:10.1002/iid3.1151)
Supplement: Supplementary file 4 — Supplementary information. [file IID3-12-e1151-s004.docx]

**Supplemental Table 4. Social demographics/social determinants per 100,000 of health of the twelve Staten Island zip codes.**

|  | **10301** | **10302** | **10303** | **10304** | **10305** | **10306** | **10307** | **10308** | **10309** | **10310** | **10312** | 10314 |
| --- | --- | --- | --- | --- | --- | --- | --- | --- | --- | --- | --- | --- |
| Age |  |  |  |  |  |  |  |  |  |  |  |  |
| Less than 19 | 26,046 | 30,778 | 33,174 | 28,291 | 23,986 | 23,565 | 21,141 | 24,235 | 26,154 | 31,159 | 24,725 | 23,908 |
| 20-44 | 35,652 | 36,599 | 36,709 | 33,297 | 34,719 | 31,433 | 34,073 | 31,100 | 35,490 | 33,643 | 32,376 | 32,248 |
| 45-65 | 26,381 | 17,723 | 23,279 | 26,182 | 28,279 | 29,364 | 27,980 | 22,389 | 22,962 | 25,639 | 29,779 | 29,028 |
| 65 and up | 11,920 | 9,273 | 6,907 | 9,148 | 13,016 | 15,638 | 9,712 | 14,892 | 10,474 | 9,559 | 13,121 | 14,816 |
| Race |  |  |  |  |  |  |  |  |  |  |  |  |
| White, Non-Hispanic | 54,392 | 49,073 | 33,861 | 47,844 | 76,244 | 87,882 | 93,431 | 93,592 | 91,058 | 54,110 | 90,817 | 75,889 |
| Black, Non-Hispanic | 24,586 | 21,008 | 37,521 | 28,841 | 4,379 | 2,112 | 610 | 515 | 2,365 | 23,291 | 970 | 4,257 |
| Asian | 6,677 | 4,264 | 7,894 | 9,547 | 10,918 | 4,548 | 2,483 | 2,917 | 3,822 | 5,560 | 5,418 | 13,448 |
| Other | 14,345 | 25,655 | 20,724 | 13,775 | 8,460 | 5,457 | 3,476 | 2,975 | 2,755 | 17,038 | 2,796 | 6,406 |
| Sex |  |  |  |  |  |  |  |  |  |  |  |  |
| Male | 48,554 | 50,320 | 47,936 | 47,889 | 48,605 | 47,735 | 49,525 | 48,573 | 50,284 | 60,324 | 48,523 | 48,110 |
| Female | 51,446 | 49,680 | 48,267 | 52,111 | 51,395 | 52,265 | 547,070 | 51,427 | 49,716 | 51,695 | 51,477 | 51,890 |
| Education status |  |  |  |  |  |  |  |  |  |  |  |  |
| Less than high school | 9,832 | 10,834 | 9,614 | 12,625 | 10,395 | 6,793 | 5,576 | 6,660 | 6,000 | 7,323 | 6,124 | 7,223 |
| High school graduate or higher | 52,257 | 45,914 | 46,744 | 52,445 | 60,569 | 63,569 | 61,237 | 68,761 | 62,078 | 54,683 | 64,999 | 64,500 |
| Bachelor's degree or higher | 20,599 | 11,903 | 12,712 | 18,188 | 22,496 | 20,823 | 21,410 | 20,971 | 21,055 | 17,086 | 22,555 | 21,932 |
| Employment status |  |  |  |  |  |  |  |  |  |  |  |  |
| Employed | 45,749 | 39,030 | 40,798 | 47,157 | 46,883 | 47,737 | 51,007 | 55,123 | 48,744 | 46,310 | 51,175 | 49,400 |
| Unemployed | 32,232 | 28,353 | 29,920 | 33,200 | 34,992 | 33,936 | 29,235 | 32,529 | 30,062 | 30,406 | 31,148 | 33,228 |
| Mean household income | $75,825 | $54,764 | $48,640 | $73,771 | $65,602 | $76,625 | $102,903 | $83,296 | $93,266 | $67,168 | $87,692 | $76,432 |
| Household income |  |  |  |  |  |  |  |  |  |  |  |  |
| Less than $25,000 | 25,150 | 27,642 | 28,024 | 23,901 | 19,863 | 17,461 | 14,229 | 11,219 | 11,119 | 25,279 | 12,531 | 15,129 |
| $25,000 - $59,999 | 26,794 | 26,001 | 27,071 | 30,425 | 24,329 | 20,607 | 18,890 | 20,393 | 21,913 | 23,927 | 21,329 | 24,005 |
| $60,000 - $149,999 | 37,393 | 34,909 | 37,447 | 34,051 | 42,537 | 42,928 | 43,600 | 45,768 | 43,975 | 35,885 | 46,444 | 44,055 |
| Greater than $150,000 | 10,663 | 11,448 | 7,457 | 11,623 | 13,271 | 19,004 | 23,281 | 22,620 | 22,992 | 14,909 | 19,696 | 16,810 |
| Means Of Transportation To Work |  |  |  |  |  |  |  |  |  |  |  |  |
| Self-transport (car, motorcycle, walk, etc.) | 32,078 | 35,262 | 33,036 | 35,290 | 37,980 | 44,395 | 55,047 | 57,061 | 50,638 | 39,764 | 51,049 | 47,604 |
| Public transport /taxicab | 24,384 | 18,782 | 19,153 | 26,452 | 22,208 | 19,043 | 11,200 | 23,535 | 16,068 | 20,180 | 14,561 | 17,384 |
| Work from home | 1,622 | 695 | 684 | 1,842 | 1,092 | 1,283 | 1,392 | 1,410 | 1,367 | 1,655 | 1,728 | 1,623 |

Data collected from the United States Postal Service, U.S. Census Bureau.^13^
